# Clinical study on acupuncture treatment of COVID-19: A protocol for a systematic review and meta-analysis

**DOI:** 10.1097/MD.0000000000028296

**Published:** 2022-01-14

**Authors:** Wenjun Luo, Yan Zhai, Mi Sun, Dong Guo, Fang Xie, Zhou Yu, Zunhao Tang

**Affiliations:** a Shandong University of Traditional Chinese Medicine, Jinan, Shandong, China; b Affiliated Hospital of Shandong University of Traditional Chinese Medicine, Jinan, Shandong, China.

**Keywords:** acupuncture, COVID-19, neurologic manifestations, protocol

## Abstract

**Background:**

COVID-19 is an acute respiratory infectious disease, which makes people difficult to breathe; in addition, it is often accompanied by headache, olfaction, and taste disorders of the neurological manifestations. Acupuncture has been proved to have a therapeutic effect on various neurologic manifestations. This study is designed to evaluate the effectiveness and safety of acupuncture for the neurologic manifestations in COVID-19.

**Methods:**

Randomized controlled trials from December 2019 to July 2021 will be included without restrictions on language or publication date. PubMed, EMBASE, Cochrane Library, Web of Science, Chinese Biomedical Databases (CBM), China National Knowledge Infrastructure (CNKI), Wanfang database, and VIP database will be searched. Two researchers will independently select studies, extract data, and evaluate study quality. Cochrane risk of bias tool for randomized trials will be used to assess the risk of bias of included studies. Statistical analyses will be performed using the Review Manager V.5.3 and stata 14.0.

**Ethics and dissemination:**

This study will not involve personal information. Ethical approval will not be required. We will publish the results in a peer-reviewed journal.

**PROSPERO Trial registration number:**

CRD42021265699

## 1 Introduction

COVID-19 is caused by severe acute respiratory syndrome coronavirus 2 (SARS-CoV-2) infection, with the characteristics of high infectivity and tall mortality, and it has rapidly developed into a worldwide public health emergency. The outbreak of the new crown pneumonia is a serious infectious disease. The novel coronavirus pneumonia patients not only show respiratory symptoms such as dyspnea, but also have nervous system symptoms such as olfaction and taste disorders, dizziness, and headache.^[1,2]^ Neurologic manifestations fell into 3 categories: central nervous system (CNS) manifestations (dizziness, headache, impaired consciousness, acute cerebrovascular disease, ataxia, and seizure), peripheral nervous system (PNS) manifestations (taste impairment, smell impairment, vision impairment, and nerve pain), and skeletal muscular injury manifestations.^[3]^ Studies have shown that the CNS and PNS are severely damaged by SARS-CoV-2, and show long-term damage.^[4]^

Acupuncture is one of the external treatments in traditional Chinese medicine with a history of more than 3000 years. Studies have proved that acupuncture has unique advantages in the treatment of some neurologic manifestations, such as neuropathic pain, olfaction, and taste disorders.^[5–8]^ Previous studies have suggested that acupuncture may work by facilitating the regeneration of olfactory neurons and regulating neuropeptides to attenuate inflammatory responses in the olfactory mucosa.^[9]^ Traditional Chinese medicine including acupuncture has played an important role in improving the neurologic manifestations and promoting recovery in patients with COVID-19.^[10,11]^

As of yet, there has been no high-quality evidence on acupuncture for the neurologic manifestations in COVID-19. Therefore, we designed this study to better understand the effectiveness and safety of acupuncture therapy for the neurologic manifestations in COVID-19.

## 2 Methods

### 2.1 Study registration

This systematic review protocol has been registered in the PROSPERO (No. CRD42021265699). We will follow recommendations outlined in The Cochrane Handbook of Systematic Review of Interventions and the Preferred Reporting Items for Systematic Reviews and Meta-Analysis Protocol (PRISMA-P) statement guidelines. If amendments are needed, we will update our protocol to include any changes in the whole process of research.

### 2.2 Types of studies

Randomized controlled trails will be included, without restrictions on language or publication date.

### 2.3 Types of participants

There are many neurologic manifestations of COVID-19, and we will choose headache, olfaction, and taste disorders as the primary presenting symptoms. Therefore, the patients should be confirmed with COVID-19 and suffer from headache and/or olfaction and/or taste disorders.

Inclusion criteria are as follows:

1. Adult with no restrictions on gender, race, disease stage.
2. Positive laboratory finding for SARS-CoV-2 or in convalescence from their COVID-19 illness
3. Subjective complaints of headache or olfaction or taste reduced olfaction after COVID-19 infection.

Exclusion criteria include history of headache or olfaction disorders or taste disorders before COVID-19 infection.

### 2.4 Types of interventions and comparisons

In addition to the treatment of COVID-19, Treatment group interventions comprised acupuncture, electro-acupuncture, auricular acupuncture, or laser acupuncture, and comparator groups intervention comprised comfort therapy (placebo, pseudo-acupuncture or blank control), other therapies (Western medicine, usual care or non-drug therapy, etc).

### 2.5 Types of outcomes

Outcomes include effectiveness indicators and safety indicators.

#### 2.5.1 Effectiveness indicators

Headache: headache frequency, headache intensity and duration of headache.

Olfaction disorders: the scores of olfactory function using validated tools such as Sniffin’ Sticks test, University of Pennsylvania Smell Identification Test, Questionnaire of Olfactory Disorders-Negative Statements, Global Rating of Smell, Global Rating of Smell Change.

taste impairment: taste acuity and discrimination.

#### 2.5.2 Safety indicator

The incidence of adverse events.

### 2.6 Search methods for identification of studies

Randomized controlled trials will be extracted from PubMed, EMBASE, Cochrane Library, Web of Science, Chinese Biomedical Databases (CBM), China National Knowledge Infrastructure (CNKI), Wanfang database, and VIP database. The complete PubMed search strategy is summarized in Table 1.

**Table 1:** PubMed search strategy.

Number	Search items
#1	“covid 19”[Title/Abstract] OR “2019-nCoV”[Title/Abstract] OR “coronavirus disease 19”[Title/Abstract] OR “2019 novel coronavirus”[Title/Abstract] OR “coronavirus disease 2019”[Title/Abstract] OR “disease 2019 coronavirus”[Title/Abstract] OR “sars coronavirus 2 infection”[Title/Abstract] OR “SARS-CoV-2”[Title/Abstract]
#2	“acupuncture”[Title/Abstract] OR “moxibustion”[Title/Abstract] OR “electroacupuncture”[Title/Abstract] OR “fire needle”[Title/Abstract] OR “acupoint injection”[Title/Abstract] OR “auricular point”[Title/Abstract] OR “warming needle moxibustion”[Title/Abstract]
#3	“Headaches”[Title/Abstract] OR “Head”[Title/Abstract] OR “Cephalodynia”[Title/Abstract] OR “Cranial”[Title/Abstract] OR “Cephalalgia”[Title/Abstract] OR “Cephalgia”[Title/Abstract] OR “olfaction” [Title/Abstract] OR “ taste disorders ” [Title/Abstract]
#4	#1 and #2 and #3

### 2.7 Data collection

#### 2.7.1 Selection of studies

**Figure 1:**
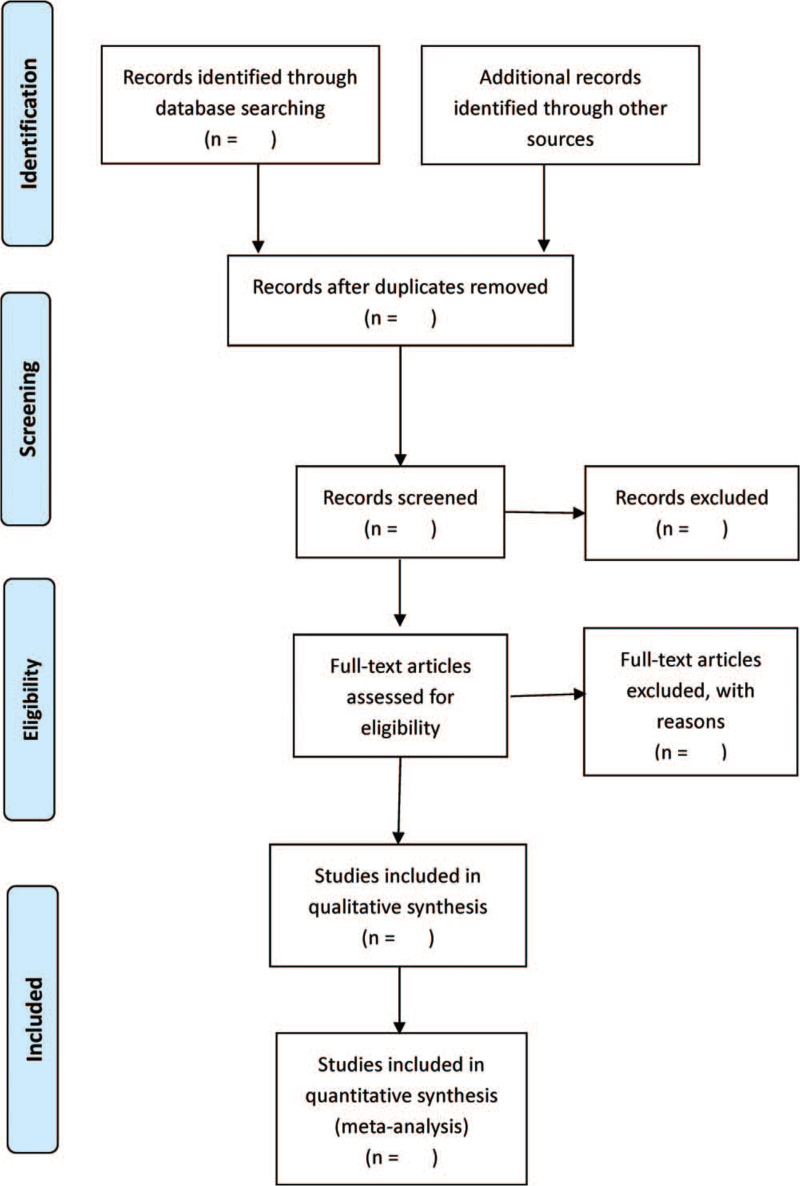
PRISMA flow diagram.

Two reviewers (WJL and YZ) will search the study independently, and then they will screen the studies by reviewing titles and abstracts or full text if necessary. Further unresolved discrepancy was managed by a third reviewer (FX). The selection process is summarized using PRISMA flow diagram. Details of the selection procedure for studies are shown in a PRISMA flow chart (Fig. 1).

#### 2.7.2 Data extraction and management

Data will then be extracted from the selected studies by 2 reviewers (ZHT and MS). The detailed extraction information are as follows: basic information of the included studies, baseline characteristics of the subjects, intervention measures and control measures, key elements of bias risk assessment, outcome indicators, etc. Any disagreements will be resolved by discussion, or by consultation with a third author (FX).

Endnote X9.3 will be used to manage the search results and perform screening and the duplicate publications will be excluded.

#### 2.7.3 Quality and bias assessment

The methodological quality and risk of bias of each trial will be assessed by 2 review authors (FX and WJL) independently according to the Cochrane Handbook. The following characteristics will be assessed: random sequence generation, allocation concealment, blinding of participants and personnel, blinding of outcome assessment, incomplete outcome data, selective reporting, and other bias. On the basis of the assessments of the studies against these 7 domains, they will be classified as being of “low risk,” “high risk,” or “unclear risk” of bias. Any disagreements will be resolved by discussion, or by consultation with another reviewer (MS).

#### 2.7.4 Dealing with missing data

If complete literature or relevant data are not available, we will contact the corresponding author. However, if the missing data cannot be obtained, then the study will be excluded from the analysis.

### 2.8 Statistical analysis

#### 2.8.1 Measures of treatment effect

Review Manager (RevMan 5.3) software will be used to conduct this meta-analysis.

Dichotomous outcomes will be presented as risk ratios (RRs) with 95% confidence intervals (95% CIs). When continuous outcomes exist, mean differences (MDs) or standardized mean differences (SMDs) will be calculated.

#### 2.8.2 Assessment of heterogeneity

The choice of whether to conduct a meta-analysis and which model to use (fixed or random effects) will depend on the level of statistical heterogeneity assessed by the *P* value and *I*^*2*^ index. *P* < .05 is considered to represent significant statistical heterogeneity, and *I*^*2*^ >50% is considered to be indicative of substantial heterogeneity.

#### 2.8.3 Data synthesis

The fixed effect model will be used if no significant heterogeneity is observed; otherwise, the random effect model will be applied for statistical analysis.^[12]^

#### 2.8.4 Subgroup analysis

According to the results of the data synthesis, we will perform subgroup analyses, or a meta-regression to analyze the source of any heterogeneity.

#### 2.8.5 Assessment of reporting bias

When outcomes include more than 10 studies, we will use Stata 14.0 to access the reporting bias by funnel plot and Egger test.^[13]^

#### 2.8.6 Sensitivity analysis

Sensitivity analyses will be performed to determine whether the results are affected by leave1out with Stata14.0.

#### 2.8.7 Quality of evidence evaluation

The evidence quality will be evaluated by 2 viewers (MS and ZY) independently with the Grading of Recommendations Assessment, Development and Evaluation (GRADE). According to 5 parameters (publication bias, indirectness, inconsistency, imprecision, and study limitations), evidence quality will be rated “high,” “moderate,” “low” according to the rating standards.

#### 2.8.8 Ethics and dissemination

As this study does not involve the patient privacy, ethical approval is not required. Our research results will be shared and shown through conference reports and peer-reviewed journals.

## 3 Discussion

Coronavirus disease in 2019 (COVID-19) is caused by severe acute respiratory syndrome coronavirus 2 (SARS-CoV-2), which has become a serious public health threat worldwide, with millions of people at risk in increasing countries.^[14]^ Dyspnea, fatigue, fever, and cough were highly prevalent in COVID-19; however, the COVID-19 pandemic has brought numerous patients complaining of neurologic manifestations, such as headache, olfaction, and taste disorders.^[15–17]^

Acupuncture was regarded as a complementary technique, and was widely applied in treating neurologic manifestations. Acupuncture is convenient, simple, and low in cost. It is benefit for COVID-19 patients with neurologic manifestations to accept acupuncture treatment. Thus, this study aimed to provide evidence of acupuncture therapy for the neurologic manifestations in COVID-19 and aided treatment decisions.

## Author contributions

**Conceptualization:** Wenjun Luo, Yan Zhai, Mi Sun, Fang Xie.

**Data curation:** Wenjun Luo, Mi Sun.

**Formal analysis:** Wenjun Luo.

**Funding acquisition:** Yan Zhai, Dong Guo.

**Investigation:** Mi Sun.

**Methodology:** Yan Zhai, Mi Sun, Fang Xie.

**Project administration:** Yan Zhai, Dong Guo, Fang Xie.

**Resources:** Mi Sun.

**Software:** Zhou Yu, Zunhao Tang.

**Supervision:** Fang Xie, Zunhao Tang.

**Validation:** Fang Xie, Zhou Yu, Zunhao Tang.

**Visualization:** Fang Xie, Zhou Yu.

**Writing – original draft:** Wenjun Luo, Mi Sun, Zhou Yu, Zunhao Tang.

**Writing – review & editing:** Wenjun Luo, Yan Zhai.
